# Study of the variation of the optical properties of calcite with applied stress, useful for specific rock and material mechanics

**DOI:** 10.1038/s41598-021-04471-6

**Published:** 2022-01-07

**Authors:** Gianfranco Ulian, Giovanni Valdrè

**Affiliations:** Centro di Ricerca Interdisciplinare di Biomineralogia, Cristallografia e Biomateriali, Dipartimento di Scienze Biologiche, Geologiche e Ambientali, Università di Bologna “Alma Mater Studiorum”, Piazza di Porta San Donato 1, 40126 Bologna, Italy

**Keywords:** Mineralogy, Optomechanics, Computational methods

## Abstract

Calcite (CaCO_3_, trigonal crystal system, space group $R\overline{3}c$) is a ubiquitous carbonate phase commonly found on the Earth’s crust that finds many useful applications in both scientific (mineralogy, petrology, geology) and technological fields (optics, sensors, materials technology) because of its peculiar anisotropic physical properties. Among them, photoelasticity, i.e., the variation of the optical properties of the mineral (including birefringence) with the applied stress, could find usefulness in determining the stress state of a rock sample containing calcite by employing simple optical measurements. However, the photoelastic tensor is not easily available from experiments, and affected by high uncertainties. Here we present a theoretical Density Functional Theory approach to obtain both elastic and photoelastic properties of calcite, considering realistic experimental conditions (298 K, 1 atm). The results were compared with those available in literature, further extending the knowledge of the photoelasticity of calcite, and clarifying an experimental discrepancy in the sign of the *p*_41_ photoelastic tensor component measured in past investigations. The methods here described and applied to a well-known crystalline material can be used to obtain the photoelastic properties of other minerals and/or materials at desired pressure and temperature conditions.

## Introduction

**Figure 1:**
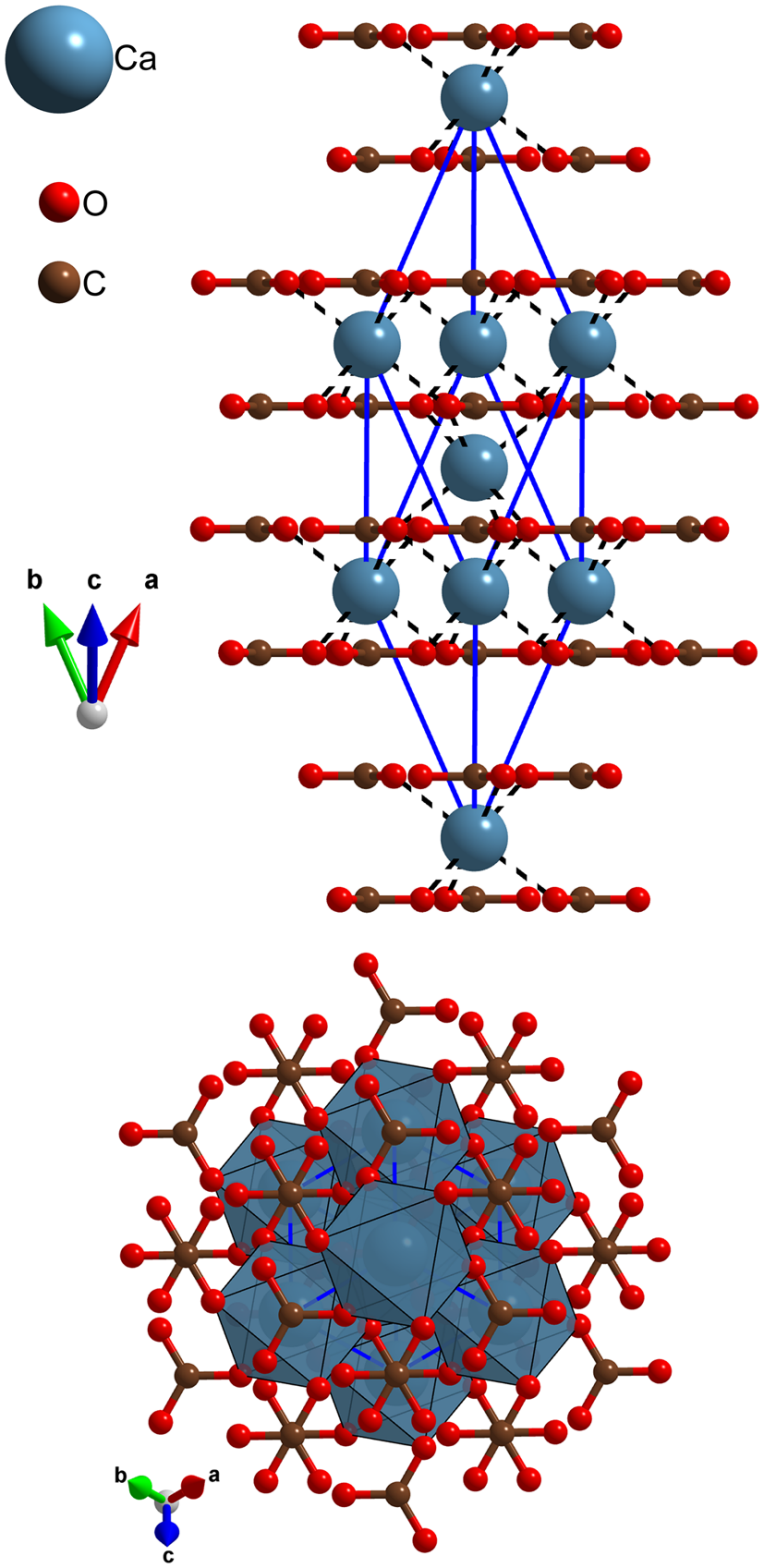
Rhombohedral cell of calcite, viewed along two crystallographic directions, [100] and [001] in the upper and lower panels, respectively. In the upper panel, the stacking of ${\mathrm{CO}}_{3}^{2-}$ and Ca^2+^ layers are clearly visible, whereas in the lower panel, the polyhedrons indicate the octahedral coordination of the Ca^2+^ ions in between the oxygen belonging to the carbonate groups.

From the mineralogical perspective, calcite (CaCO_3_, space group $R\overline{3}c$, rhombohedral-I class) is an anisotropic crystalline phase formed by layers of ${\mathrm{CO}}_{3}^{2-}$ anions, with covalent C–O bonds, alternately stacked to layers of Ca^2+^ ions held together by ionic interactions^1^. In addition, this mineral can be considered a model of heterodesmic structure, because it presents different types of bonds between the atoms and/or atomic groups. A graphical representation of the crystal structure of calcite is presented in Fig. 1.

Due to its structural, elastic, and optical properties, calcite is an important mineral in the scientific and technological fields, with wide applications in mineralogy, geology, chemistry, materials science, optics, and instrumentation engineering^2–4^. Calcite is one of the most birefringent materials (*δ* = 0.172, with *δ* being birefringence), where its transparent variety is known as *Iceland spar*. In general, birefringence derives from the anisotropic refractive indexes of the mineral, which in turn are related to its dielectric (second-rank) tensor. When a crystal is deformed by a stress (described with a second-rank tensor), its optical properties change according to the photoelastic (or elasto-optics, **p**) and piezo-optics (**π**) fourth-rank tensors, which correlate the variations of the inverse of the dielectric tensor with the applied deformation. The relationship between the second-rank stress tensor **σ** to the second-rank pure strain tensor **η** is given by the generalized Hooke’s law,

1 $$ \sigma_{ij} = C_{ijkl} \eta_{kl} $$

and

2 $$ \eta_{ij} = S_{ijkl} \sigma_{kl} $$

where *C*_*ijkl*_ and *S*_*ijkl*_ are the components of the fourth-rank stiffness and compliance tensors **C** and **S**, respectively, with **S** = **C**^–1^. The indexes *i*, *j*, *k* and *l* represent Cartesian directions *x*, *y* and *z*. Both stress and strain tensors are symmetric, hence their independent components are 6 and the 81 elastic components reduces to 36. By using the Voigt’s notation, pairs of Cartesian indexes can be mapped by a single index, so that just two indexes *v, u* = 1, …, 6 can be used, where 1 = *xx*, 2 = *yy*, 3 = *zz*, 4 = *yz*, 5 = *xz* and 6 = *xy*^5^. With this notation, the fourth-rank elastic tensor **C** is represented by a symmetric 6 × 6 matrix and the stress–strain relation can be rewritten as $\sigma_{v} = C_{vu} \eta_{u}$ and $\eta_{v} = S_{vu} \sigma_{u}$. In terms of crystal lattice, the components of the stiffness matrix are defined as

3 $$ C_{vu} = \left( {{{\partial^{2} E} \mathord{\left/ {\vphantom {{\partial^{2} E} {\partial \eta_{v} \partial \eta_{u} }}} \right. \kern-\nulldelimiterspace} {\partial \eta_{v} \partial \eta_{u} }}} \right)\Omega^{ - 1} $$

with Ω the unit cell volume and *E* the energy of the system. This relationship shows that the terms *C*_*vu*_ and *C*_*uv*_ are equivalent, resulting in the symmetry of the **C** and **S** matrices and in the further reduction from 36 to 21 independent elastic components.

For all but isotropic (cubic) crystalline materials, the dielectric constants are described by a second-rank tensor $\epsilon$, which determines the optical indicatrix of the system. In the case of calcite, the optical indicatrix is negative, because the extraordinary refractive index, *n*_*ε*_*,* is lower than the ordinary one, *n*_*ω*_. When the crystal is strained, the variation of the inverse dielectric tensor $\epsilon^{ - 1}$ can be related to the amount of induced strain by means of the fourth-rank photoelastic (Pöckels’) tensor **p**, whose components are known as elasto-optic or strain-optical coefficients. Hence, the elasto-optic constants can be calculated as

4 $$ p_{ijkl} = {{\partial \Delta  \epsilon_{ij}^{ - 1} } \mathord{\left/ {\vphantom {{\partial \Delta  \epsilon_{ij}^{ - 1} } {\partial \eta_{kl} }}} \right. \kern-\nulldelimiterspace} {\partial \eta_{kl} }} $$

where $\Delta  \epsilon_{ij}^{ - 1}$ are the differences between the strained and unstrained inverse dielectric tensor. As in the case of the elastic tensor, it is possible to reduce the fourth-rank Pöckels’ tensor to a 6 × 6 matrix, where $p_{vu} = {{\partial \Delta  \epsilon_{v}^{ - 1} } \mathord{\left/ {\vphantom {{\partial \Delta  \epsilon_{v}^{ - 1} } {\partial \eta_{u} }}} \right. \kern-\nulldelimiterspace} {\partial \eta_{u} }}$. Given the stress–strain relationship previously introduced, the piezo-optic tensor **π**, *i.e.*, the fourth-rank tensor whose *π*_*vu*_ components correlate the $\Delta  \epsilon_{v}^{ - 1}$ variations with the stress *σ*_*u*_, can be obtained with

5 $$ \pi = {\mathbf{pS}} $$

and

6 $$ {\mathbf{p}} = \pi {\mathbf{C}}. $$

It is worth mentioning that, although the elastic tensor of a material is symmetric, the photoelastic one is generally not, thus *p*_*vu*_ ≠ *p*_*uv*_ and *π*_*vu*_ ≠ *π*_*uv*_ and both **p** and **π** have 36 independent components. This knowledge finds its usefulness in the determination of the stress state of rocks and materials and/or for the analysis of fractures in rock samples containing calcite, if the photoelastic and/or piezo-optic properties are accurate enough^6,7^.

The photoelasticity of calcite was experimentally determined and widely published in the past literature, where we observed and reviewed quite scattered results, with the same photoelastic tensor component showing high variation up to about 200%, leaving the reader doubtful and confused (see for instance refs^8–12^). This wide range and scattering of results is associated to the experimental methods employed to characterize photoelasticity and piezo-optics of materials, which are complex and involve the measurements of different quantities, e.g., elastic properties and refractive indexes, on specifically cut and prepared samples^13–15^. It must be also emphasized that the just cited properties were determined in several studies carried out by various researchers, who performed the measurements in different experimental conditions and instrumental setups, resulting in scattered data with different accuracies^16^. For example, the calcite photoelasticity was measured by variations of refractive index^8,9^, Brillouin scattering^10^ and analysis of Raman spectral intensities^12^, where each technique presented different degrees of accuracy. In fact, as also discussed by Andrushchak and co-workers^17^, some of the employed experimental means (e.g., Brillouin scattering techniques) are accompanied by very high uncertainties, which often lead to ill-defined photoelastic/piezo-optic constants, in particular on their sign. Indeed, this is the case of the photoelastic tensor component *p*_41_ of calcite, whose sign disagrees between the different experiments as reported in the references^10,18^.

Here, we propose a theoretical (Density Functional Theory, DFT) investigation of the photoelastic and piezo-optic properties of calcite to further extend the knowledge of the optical properties of this material, and to aid answering the above cited uncertainties and cross-correlating the simulated and experimental data. This work aims also at showing the interested reader a possible way to calculate and model this physical property for other minerals and/or materials of interest for both mineralogical, geological and materials science applications.

### Structural and dielectric properties of calcite

We performed first-principle DFT simulations to calculate the photoelastic properties of calcite, both at absolute zero (standard DFT settings) and in typical experimental conditions (298 K, 1 atm), using two well-known approaches, PBE-D2 and B3LYP-D*, which include the effects of long-range interactions (see the Methods section for details).

The necessary starting point to calculate the photoelastic properties of calcite is given by a good description of both the crystal structure and the dielectric properties. The simulation results related to the equilibrium geometry of calcite are reported in Table 1. It can be noted that the inclusion of long-range interactions via a semi-empirical scheme produced a unit cell that is closer to the X-ray diffraction refinement found in literature^19^ and reported in Table 1. At 298 K and with the inclusion of van der Waals correction, the unit cell volume is in line with that of the XRD refinement at the same conditions, with only a slight overestimation for both functionals (ΔΩ_PBE-D2_ =  + 2.0%, ΔΩ_B3LYP-D*_ =  + 2.3%). To our knowledge, this is the first time that a proper treatment for long-range interactions is included in the calculation of the total DFT energy for calcite at room temperature. As expected, because of the heterodesmic nature of calcite, the dispersive forces are more effective on the *c* lattice parameter than on the *a* = *b* one, since in the trigonal unit cell of the mineral there are layers of Ca^2+^ ions alternately stacked (along the *z* direction) with layers of CO_3_^2–^ anions (see Fig. 1). In this regard, the proposed approach including the dispersive forces correctly describes the Ca^2+^–O^2–^ attractive interactions, resulting in an optimized distance between the layers, and gives a correct description of the unit cell of a crystal, which is mandatory for obtaining reliable structure-dependent properties, such as vibrational^20,21^, elastic^22^ and photoelastic ones^23^.

**Table 1:** Calculated structure (unit cell parameters *a* and *c*, volume Ω, C–O bond distance *d*_C–O_ and Ca–O interaction distance *d*_Ca–O_), dielectric tensor components (static $\epsilon^{0}$ and high-frequency $\epsilon^{\infty }$), refractive index (ordinary *n*_*ω*_ and extraordinary *n*_*ε*_) and birefringence (*δ*) of calcite, obtained at DFT level with PBE-D2 and B3LYP-D* functionals corrected for long-range interactions in static condition (0 K) and at 298 K (at 1 atm = 0.0001 GPa).

	Experimental	PBE^c^	PBE-D2^d^	PBE-D2^e^	B3LYP^c^	B3LYP-D* ^d^	B3LYP-D*^e^
*a* (Å)	4.991^a^	5.039	5.020	5.032	5.037	5.028	5.041
*c* (Å)	17.062^a^	17.402	16.955	17.118	17.330	16.968	17.116
Ω (Å^3^)	368.1^a^	382.7	370.1	375.4	380.8	371.5	376.7
ΔΩ (%)	0^a^	+ 4.0	+ 0.5	+ 2.0	+ 3.5	+ 0.9	+ 2.3
*d*_C–O_ (Å)	1.2840^a^	1.2978	1.2952	1.2963	1.2878	1.2860	1.2870
*d*_Ca–O_ (Å)	2.3590^a^	2.3900	2.3607	2.3733	2.3907	2.3695	2.3818
$\epsilon_{xx}^{0}$	8.5^b^	–	11.26	10.53	7.8	8.54	8.40
$\epsilon_{zz}^{0}$	8.0^b^	–	9.15	8.11	6.4	7.50	7.00
$\epsilon_{xx}^{\infty }$	2.7^b^	–	2.80	2.78	2.6	2.59	2.57
$\epsilon_{zz}^{\infty }$	2.2^b^	–	2.20	2.19	2.1	2.08	2.06
*n*_*ω*_	1.640–1.660^b^	–	1.674	1.668	–	1.609	1.603
*n*_*ε*_	1.486^b^	–	1.483	1.479	–	1.441	1.437
*Δ*	0.1540–0.1740^b^	–	0.191	0.189	–	0.168	0.166

^a^^19^; ^b^^24^; ^c^^20^; ^d^Present work (0 K); ^e^Present work (298 K).

Table 1 reports also the static and high-frequency dielectric constants ($\epsilon^{0}$ and $\epsilon^{\infty }$, respectively), the refractive index *n* and the birefringence *δ*. At ambient conditions (298 K, 1 atm), the components of the static dielectric tensor along the *xx* and *zz* directions are in good agreement with the experimental and theoretical ones tabulated in literature ($\epsilon_{xx}^{0}$ = 8.5, $\epsilon_{zz}^{0}$ = 8.0)^24,25^. In general, PBE-D2 data are slightly higher than those calculated at B3LYP-D* level, with the maximum difference of ca. + 18% on $\epsilon_{xx}^{0}$.

### Elastic and photoelastic properties of calcite

We report in Table 2 the elastic (*C*_*vu*_), photoelastic (*p*_*vu*_) and piezo-optic (*π*_*vu*_) constants calculated at PBE-D2 and B3LYP-D* level of theory. Let us discuss first the elastic moduli, where it can be noted that at 298 K both functionals corrected for the long-range interactions provide a good description of the stiffness tensor components, with respect to the experimental results of Dandekar^26^ and Chen and co-workers^27^, with values slightly increased by about 10%. In fact, it is well-known that, even by including the thermal effects, there is a small overestimation of the elastic moduli because of the Pulay stress, i.e., an effect due to the incompleteness of the basis sets. This effect occurs during the derivation of the basis sets with respect to the position of the atoms and slightly increases the values of the elastic tensor components.

**Table 2:** Calcite elastic (*C*_*vu*_, GPa), photoelastic (*p*_*vu*_, dimensionless) and piezo-optic (*π*_*vu*_, TPa^–1^) components of the corresponding fourth-rank tensors, expressed in Voigt’s notation.

	Elastic constants							
	*C*_11_	*C*_33_	*C*_12_	*C*_13_	*C*_14_	*C*_44_	*C*_66_	
PBE-D2 (0 K)	161.47	84.01	66.09	59.75	– 20.80	35.64	47.69	
PBE-D2 (298 K)	154.06	80.92	61.56	55.29	– 19.02	33.48	46.25	
B3LYP-D* (0 K)	163.94	89.65	63.75	59.43	– 20.60	37.35	50.09	
B3LYP-D* (298 K)	156.47	86.03	59.85	55.13	– 18.86	35.24	48.31	
Experimental (298 K)^a^	146.3	85.3	59.7	50.8	– 20.8	34.0	43.3	
Experimental (298 K)^b^	149.4	85.2	57.9	53.5	– 20.0	34.1	45.8	

	Photoelastic constants							
	*p*_11_	*p*_12_	*p*_13_	*p*_14_	*p*_31_	*p*_33_	*p*_41_	*p*_44_
PBE-D2 (0 K)	0.100	0.180	0.215	– 0.026	0.267	0.159	– 0.052	– 0.054
PBE-D2 (298 K)	0.104	0.179	0.215	– 0.026	0.269	0.159	– 0.058	– 0.052
B3LYP-D* (0 K)	0.113	0.190	0.224	– 0.018	0.266	0.169	– 0.050	– 0.053
B3LYP-D* (298 K)	0.116	0.190	0.225	– 0.022	0.267	0.170	– 0.054	– 0.051
Experimental (296 K)^c^	0.062	0.147	0.186	– 0.011	0.241	0.139	– 0.036	– 0.058
Experimental (298 K)^d^	0.095	0.189	0.215	– 0.006	0.309	0.178	0.010	–0.09
Experimental (298 K)^e^	-	-	0.346	– 0.011	0.113	0.224	– 0.002	– 0.058

	Piezo-optic constants							
	*π*_11_	*π*_12_	*π*_13_	*π*_14_	*π*_31_	*π*_33_	*π*_41_	*π*_44_
PBE-D2 (0 K)	– 0.861	0.685	2.683	– 1.628	1.082	0.349	– 1.181	– 2.895
PBE-D2 (298 K)	– 0.784	0.705	2.716	– 1.636	1.145	0.400	– 1.230	– 2.949
B3LYP-D* (0 K)	– 0.606	0.649	2.473	– 1.184	1.034	0.515	– 1.027	– 2.562
B3LYP-D* (298 K)	– 0.577	0.701	2.537	– 1.322	1.088	0.585	– 1.064	– 2.584
Experimental (296 K)^c^	– 0.85	0.65	2.30	– 1.29	1.14	0.18	– 1.10	– 3.18
Experimental (298 K)^d^	– 0.60	0.90	2.48	– 1.09	1.55	0.44	– 0.66	– 3.40

^a^^26^, ^b^^27^, ^c^^10^, ^d^^9^, ^e^^11^, calculated using results from Nelson et al.^10^.

The elastic constants *C*_11_, *C*_33_, *C*_44_ and *C*_66_ obtained with the hybrid B3LYP functional are about 2–6% higher than those calculated with PBE, while the other stiffness components show an underestimation of about 1%. This is a common figure of the two approaches because the standard generalized-gradient approximation functional is associated to both underbinding between atoms and underestimated bulk moduli for ionic compounds, as recently shown by Zhang and co-workers^28^. For solids as calcite, the use of hybrid functionals that include a fraction of exact Fock exchange is preferred because they are more accurate in the simultaneous description of both structural (lattice) and energy (e.g., cohesive) properties^22,28,29^.

The photoelastic *p*_*vu*_ and piezo-optic *π*_*vu*_ components obtained from our DFT simulations (at 0 K and 298 K) for *ω* = 0 are reported in Table 2, together with those obtained by different experimental measurements^9–12^. In the next paragraphs, we discuss the present simulation results with those experimentally derived by Pöckels^9^ and Nelson et al.^10^.

**Figure 2:**
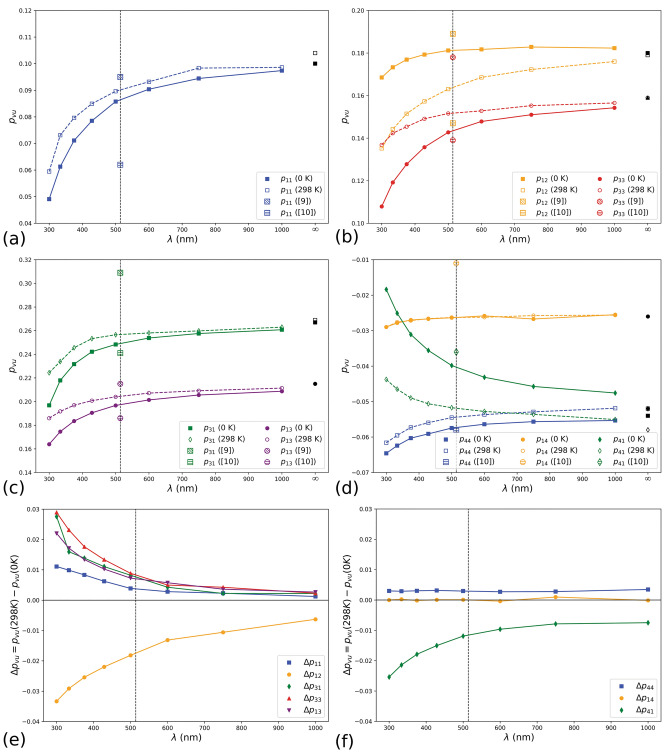
The independent Pöckels’ tensor components of calcite calculated at finite wavelengths *λ* at the DFT/PBE-D2 level of theory, (**a**) *p*_11_, (**b**) *p*_12_ and *p*_13_, (**c**) *p*_31_ and *p*_13_ and (**d**) *p*_44_, *p*_14_, *p*_41_. The lines connecting the points are meant as a guide for the eye, whereas the dashed vertical line is the *λ* = 514 nm used in the experiments of Pöckels^9^ and Nelson et al.^10^, whose respective data at such wavelength are reported together with the theoretical ones. The black symbols are the photoelastic constants calculated in our simulations in static limit (*λ* = ∞). Panels (**e**) and (**f**) report the differences Δ*p*_*vu*_ between the calculated room temperature and 0 K photoelastic constants as a function of λ.

Our theoretical study suggests that the *p*_41_ photoelastic constant is negative in the selected crystal orientation (crystallographic **a**-axis and **c-**axis parallel to the – *x* and *z* Cartesian directions, respectively). As also suggested by Erba and Dovesi^23^, this discrepancy on the sign of the photoelastic component could arise from the finite wavelength of the laser sources used in the different experimental studies. Indeed, our theoretical photoelastic and piezo-optic constants of calcite reported in Table 2 were obtained at zero electric field frequency (*ω* = 0, *λ* = ∞), but it is known that the experiments generally employ monochromatic lasers centred at a specific wavelength, hence the experimentally derived photoelasticity does not correspond to that of the static limit. To check if the finite wavelength could be the same source of discrepancy experimentally observed in calcite, and at the same time to show how it affects the *p*_*vu*_ components, we calculated the fourth-rank tensor **p** at different *λ* between 300 and 1000 nm (hence, at electric field frequency *ω* ≠ 0) at the PBE-D2 level of theory, both at room temperature (298 K) and in static conditions (0 K). The results are graphically reported in Fig. 2, showing that all but the *p*_14_ tensor component have a dependence on the wavelength λ.

In detail, there is a strong variation of the strain-optical components for 300 nm < *λ* < 500 nm, then the *p*_*vu*_ tensor components asymptotically reach the value calculated at the λ = ∞ limit, with a trend of the form $p_{vu} \left( \lambda \right) = {{ - k} \mathord{\left/ {\vphantom {{ - k} \lambda }} \right. \kern-\nulldelimiterspace} \lambda } + p_{vu} \left( \infty \right)$ within the 300 nm–1000 nm range. The *p*_41_ value as a function of *λ* is always lower than zero (Fig. 2b), and no change of sign in the explored wavelength range was observed. Hence, this analysis showed that for calcite the experimental ambiguity of the sign of the *p*_41_ photoelastic component is not related to the laser source employed in the experiments, but it could be related to some instrumental uncertainties or in the orientation of the crystal during the measurements carried out by Pöckels^9^. Similar discussions were proposed in a relatively recent review by Mytsyk^30^, who explained that for some crystal classes (such as $\overline{3} m$) the choice of the reference system can influence the sign of shifting and rotating piezo-optic components.

Most of the *p*_*vu*_ components show a variation between the values calculated at room and absolute zero temperatures, Δ*p*_*vu*_ = *p*_*vu*_(298 K) – *p*_*vu*_(0 K) (Fig. 2c,d), which asymptotically reaches zero as a function of *λ*. The calculated Δ*p*_*vu*_ are in absolute terms higher for *λ* < 500 nm. The exceptions to this trend are given by the *p*_14_ and *p*_44_ components, whose temperature differences are almost constants (Fig. 2d).

Our work provides the first ab initio determination of the photoelastic and piezo-optic tensor components at 0 K and at 298 K of calcite, together with their related quantities (crystal structure, optical and elastic properties), showing the relevancy of the use of quantum mechanical methods in this research field. The **p** and **π** tensors are very stable with respect to internal DFT parameters, meaning that theoretical simulations performed within this framework are suitable for this kind of characterization. This work further extends the knowledge of important physical properties of anisotropic and heterodesmic crystals, providing a complete description as a function of *λ* of the photoelastic (strain dependent) and piezo-optical (stress dependent) response of calcite, which can be employed to analyse the state of stress and strain within a geological sample and/or a material and envisage other possible applications. In addition to being useful to clarify potential ambiguities in the sign of photoelastic/piezo-optic constants determined experimentally, the theoretical approach here exploited is indeed extremely convenient and useful to cross-check the data, because experimental acousto-optical techniques are cumbersome and complex. In fact, Andrushchak and collaborators^16^ showed that with optical interferometry 57 measurements on 16 specifically cut samples are necessary to obtain the 36 independent photoelastic constants, and, for a trigonal crystal of the $\overline{3} m$ class, 11 measurements on 2 samples are still needed. The present approach could be extremely useful also to model the photoelastic and piezo-optic behaviour of other anisotropic crystals, such as those recently developed by Tear and co-workers^31,32^.

## Methods

### Computational approach

In the present work, the investigation of the photoelasticity of calcite was conducted within the Density Functional Theory (DFT) framework, employing the CRYSTAL17 code^33^. Regarding the Hamiltonian, we used both the standard generalized gradient approximation (GGA) functional developed by Perdew–Burke–Ernzerhof (PBE)^34^, and the hybrid B3LYP^35,36^. We studied the effects of different DFT functionals on the calculated properties of the material and, in this case, both PBE and B3LYP are well-established functionals for solid state physics applications. However, a typical drawback of the selected Hamiltonians is the lack of adequate treatment of long-range interactions, which we here accounted for by means of the DFT-D2 scheme^37^. To this purpose, we employed a specific parametrization of the selected correction for dispersive forces for B3LYP, labelled as B3LYP-D*^38^. The multi-electron wave function was constructed using a linear combination of atomic orbitals, which in turn are described as Gaussian-type functions. We employed for calcite a basis set that was optimized in the previous work of Valenzano and co-workers^20^, more specifically the one labelled as BSD (basis set D) in the cited work. The total energy was calculated by sampling the First Brillouin zone with a 6 × 6 × 6 Monkhorst–Pack mesh (32 irreducible *k*-points), setting the tolerances for the self-consistent field loop to 10^–8^ Ha.

### Dielectric tensor

The electronic dielectric tensors at equilibrium, $\vec{ \epsilon }\left( 0 \right)$, and at each deformation step, $\vec{ \epsilon }\left( \eta \right)$, were calculated according to the following expression reported by Erba and Dovesi^23^:

7 $$ \vec{ \epsilon } = 1 + \frac{4\pi }{\Omega }\vec{\alpha } $$

with $\vec{\alpha }$ and Ω being the electronic polarizability tensor and the unit cell volume, respectively. The polarizability is evaluated analytically with a coupled-perturbed Hartree–Fock/Kohn–Sham (CPHF/KS) approach adapted for solid systems as described by Ferrero and co-workers^39–42^. It is a self-consistent, perturbative method that describes the relaxation of the crystalline orbitals when affected by an external electric field $\vec{E}$. Taking the electronic charge as *e* = – 1 a.u., and as indicated by Gu et al.^43^, the general expression of the Hamiltonian $\hat{H}$ from this approach is given by:

8 $$ \hat{H} = \hat{H}_{0} + \hat{H}^{\prime} = \hat{H}_{0} + \vec{E} \cdot \vec{r} = \hat{H}_{0} + \left( {\vec{E}_{st} + 2\vec{E}_{\omega } \cos m\omega t} \right) \cdot \vec{r} $$

where $\hat{H}_{0}$ and $\hat{H}^{\prime}$ are the unperturbed and perturbed Hamiltonian operators, respectively, the latter being the sum of the static electric field, $\vec{E}_{st}$ (frequency *ω* = 0, wavelength *λ* = ∞), and the frequency-dependent one, whose amplitude and frequency of oscillation are $\vec{E}_{\omega }$ and *ω*, respectively. $\vec{r}$ is the electron position vector in real space. However, the scalar potential operator $\hat{H}^{\prime} = \vec{E} \cdot \vec{r}$ is unbounded and breaks the translational symmetry, hence, for periodic structures, it is replaced by the following formula, as suggested by Maschio et al.^44^:

9 $$ \hat{H}^{\prime} = i\vec{E} \cdot e^{{i\vec{k} \cdot \vec{r}}} \vec{\nabla }_{k} e^{{ - i\vec{k} \cdot \vec{r}}} = \sum\limits_{b} {E_{b} (t)\hat{h}^{{\left( {E_{b} (t)} \right)}} (\vec{k})} $$

with $\vec{k}$ indicating a reciprocal space vector, *E*_*b*_(*t*) and $\hat{h}^{{\left( {E_{b} (t)} \right)}}$ being the *b* component of the applied electric field and of the gradient vector, respectively. The total energy of the system, *E*_tot_, under the effect of the electric field perturbation can then be expressed in terms of a perturbative series of the field components, indicated with the indexes *i*, *j* and *k*:

10 $$ E_{tot} = E_{tot}^{\left( 0 \right)} - \sum\limits_{i} {\mu_{i} \vec{E}_{i} } - \frac{1}{2!}\sum\limits_{i,j} {\alpha_{ij} \vec{E}_{i} \vec{E}_{j} } - \frac{1}{3!}\sum\limits_{i,j,k} {\beta_{ijk} \vec{E}_{i} \vec{E}_{j} \vec{E}_{k} } + \ldots $$

where $E_{tot}^{\left( 0 \right)}$ is the unperturbed total energy and the tensors of increasing rank *μ*, *α* and *β* are the permanent electric dipole moment, the polarizability and the hyperpolarizability, respectively, as reported by Ferrero et al.^45^. The interested reader can find in dedicated literature^43–46^ all the information related to CPHF/KS approach for periodic systems, and the detail of its implementation in the CRYSTAL code^39–42^, which allows calculating the electronic contribution to the dielectric tensor for both static (frequency *ω* = 0, wavelength *λ* = ∞) and frequency-dependent (*ω* ≠ 0, *λ* ≠ ∞) electric fields.

### Elastic and photoelastic constants

The elastic moduli *C*_*vu*_ and photoelastic *p*_*vu*_ constants were calculated using the following three independent strains of the unit cell, i.e., *η*_1_ = *η*_2_, *η*_3_ and *η*_4_ = *η*_5_ = *η*_6_, which were sufficient to obtain each independent elastic and photoelastic component for a rhombohedral-I crystal class^23,47^:

11 $$ \eta_{1} = \left( {\begin{array}{*{20}c} \delta & 0 & 0 \\ 0 & 0 & 0 \\ 0 & 0 & 0 \\ \end{array} } \right),\;\eta_{3} = \left( {\begin{array}{*{20}c} 0 & 0 & 0 \\ 0 & 0 & 0 \\ 0 & 0 & \delta \\ \end{array} } \right),\;\eta_{4} = \left( {\begin{array}{*{20}c} 0 & 0 & 0 \\ 0 & 0 & \delta \\ 0 & \delta & 0 \\ \end{array} } \right) $$

For each independent strain, we employed 5 deformations with strain amplitude *δ* ± 0.010 Å (step of 0.005 Å). The internal geometry (atomic positions) was optimized at fixed lattice parameters to include the nuclear relaxation, and then it was calculated the electronic dielectric tensors at both equilibrium, and strained configuration, $\vec{ \epsilon }\left( 0 \right)$ and $\vec{ \epsilon }\left( \eta \right)$, respectively^23^.

The crystallographic axes of calcite were oriented with the crystallographic **a**-axis and **c-**axis parallel to the – *x* and *z* Cartesian directions, respectively. Within this convention, and according to Nye^5^, the independent and non-zero components of the stiffness tensor **C** for a crystal of rhombohedral-I class are:

12 $$ {\mathbf{C}} = \left( {\begin{array}{*{20}c} {C_{11} } & {C_{12} } & {C_{13} } & { - C_{14} } & \cdot & \cdot \\ {} & {C_{11} } & {C_{13} } & {C_{14} } & \cdot & \cdot \\ {} & {} & {C_{33} } & \cdot & \cdot & \cdot \\ {} & {} & {} & {C_{44} } & \cdot & \cdot \\ {} & {} & {} & {} & {C_{44} } & { - C_{14} } \\ {} & {} & {} & {} & {} & {C_{66} } \\ \end{array} } \right) $$

where the dots in the 6 × 6 matrix are null elements and with *C*_66_ = (*C*_11_ – *C*_12_)/2. Similarly, we can define the photoelastic (Pöckels’) tensor **p** as:

13 $$ {\mathbf{p}} = \left( {\begin{array}{*{20}c} {p_{11} } & {p_{12} } & {p_{13} } & { - p_{14} } & \cdot & \cdot \\ {p_{12} } & {p_{11} } & {p_{13} } & {p_{14} } & \cdot & \cdot \\ {p_{31} } & {p_{31} } & {p_{33} } & \cdot & \cdot & \cdot \\ { - p_{41} } & {p_{41} } & \cdot & {p_{44} } & \cdot & \cdot \\ \cdot & \cdot & \cdot & \cdot & {p_{44} } & { - p_{41} } \\ \cdot & \cdot & \cdot & \cdot & { - p_{14} } & {p_{66} } \\ \end{array} } \right) $$

where *p*_66_ = (*p*_11_ – *p*_12_)/2. By previous definition, the piezo-optic tensor **π** has the same structure as that of the photoelastic components:

8 $$ {{\varvec{\uppi}}} = \left( {\begin{array}{*{20}c} {\pi_{11} } & {\pi_{12} } & {\pi_{13} } &\vline & { - \pi_{14} } & \cdot & \cdot \\ {\pi_{12} } & {\pi_{11} } & {\pi_{13} } &\vline & {\pi_{14} } & \cdot & \cdot \\ {\pi_{31} } & {\pi_{31} } & {\pi_{33} } &\vline & \cdot & \cdot & \cdot \\ \hline { - \pi_{41} } & {\pi_{41} } & \cdot &\vline & {\pi_{44} } & \cdot & \cdot \\ \cdot & \cdot & \cdot &\vline & \cdot & {\pi_{44} } & { - \pi_{41} } \\ \cdot & \cdot & \cdot &\vline & \cdot & { - \pi_{14} } & {\pi_{66} } \\ \end{array} } \right) = \left( {\begin{array}{*{20}c} {\mathbf{A}} &\vline & {\mathbf{B}} \\ \hline {\mathbf{C}} &\vline & {\mathbf{D}} \\ \end{array} } \right) $$

As shown in the above formulation, the piezo-optic matrix **π** can be subdivided in four 3 × 3 sub-matrices A, B, C and D. When the indexes *v, u* = 1, 2, 3, the *π*_*vu*_ components are called the *principal* coefficients, which describe the relation between the principal refractive indexes and the normal stresses (sub-matrix **A**). For *v* = 1, 2, 3 and *u* = 4, 5, 6 (sub-matrix **B**), the piezo-optic components are referred as the *shifting* coefficients, connecting the variations of the principal refractive indexes with shear stresses. Sub-matrices **C** (*v* = 4, 5, 6 and *u* = 1, 2, 3) and **D** (*v, u* = 4, 5, 6) contain the so-called *rotating* and *rotating-shifting* piezo-optic components, which describe the rotation of the optical indicatrix under the effect of normal and shear stresses, respectively^17^.

The calculation of the structural, dielectric and (photo)elastic properties were conducted both in static conditions, i.e., at 0 K without any thermal contribution, and at room temperature (298 K) at a pressure of 1 atm (= 0.0001 GPa). For the latter, we employed the quasi-harmonic approximation to introduce the temperature effect on the cited properties, as described in previous literature^48–50^. Five unit cell volumes, the equilibrium one, two compressed and two expanded, were used for the quasi-harmonic approximation. Finally, we employed the so-called “quasi-static approximation (QSA)” to describe the thermo-elasticity of calcite, which assumes that the stiffness depends only on the thermal expansion of the crystal as suggested by Destefanis et al.^51^, where, as explained, it was shown that QSA provides a qualitatively good description of the thermo-elastic constants.

## References

[1] Effenberger H, Zemann J, Mereiter K (1981). Crystal structure refinements of magnesite, calcite, rhodochrosite, siderite, smithonite, and dolomite, with discussion of some aspects of the stereochemistry of calcite type carbonates. Z Kristallogr. New Cryst. Struct..

[2] Friese MEJ, Nieminen TA, Heckenberg NR, Rubinsztein-Dunlop H (1998). Optical alignment and spinning of laser-trapped microscopic particles. Nature.

[3] Herne CM, Lyons FE, Galvez EJ, Sam A, Dholakia K, Spalding GC (2020). Polarimetry studies on birefringent materials in optical tweezers. SPIE.

[4] Kang SS, Nakamura N, Fukuda K, Oikawa Y, Obara Y (2000). Interpretation on stress history of Torigata limestone deposit based on rock stresses measured by three methods. Mem. Fac. Eng. Kumamoto Univ..

[5] Nye JF (1957). Physical Properties of Crystals.

[6] Noselli G, Dal Corso F, Bigoni D (2010). The stress intensity near a stiffener disclosed by photoelasticity. Int. J. Fract..

[7] Uenishi K (2015). Dynamic dip-slip fault rupture in a layered geological medium: Broken symmetry of seismic motion. Eng. Fail. Anal..

[8] Pockels F (1889). Ueber den einfluss elastischer deformationen, speciell einseitigen druckes, auf das optische verhalten krystallinischer körper. Ann. Phys..

[9] Pockels F (1903). Ueber die aenderung des optischen verhaltens verschiedener gläser durch elastische deformation. Ann. Phys..

[10] Nelson DF, Lazay PD, Lax M (1972). Brillouin scattering in anisotropic media: calcite. Phys. Rev. B.

[11] Kumari GS, Rao NR (1983). Photoelastic constants of calcite from its first-order Raman spectrum. Phys. Rev. B.

[12] Kumari GS, Rao NR (1985). Erratum: Photoelastic constants of calcite from its first-order Raman spectrum (Physical Review B (1985) 32, 6, (4239–4240)). Phys. Rev. B.

[13] Montalto L, Natali PP, Scalise L, Paone N, Davì F, Rinaldi D (2019). Quality control and structural assessment of anisotropic scintillating crystals. Curr. Comput. Aided Drug Des..

[14] Scafidi M, Pitarresi G, Toscano A, Petrucci G, Alessi S, Ajovalasit A (2015). Review of photoelastic image analysis applied to structural birefringent materials: Glass and polymers. Opt. Eng..

[15] Umezaki E (2013). Stress distribution measurement techniques using photoelasticity: Current status and future prospects. Seimitsu Kogaku Kaishi.

[16] Erba A, Ruggiero MT, Korter TM, Dovesi R (2015). Piezo-optic tensor of crystals from quantum-mechanical calculations. J. Chem. Phys..

[17] Andrushchak AS, Bobitski YV, Kaidan MV, Mytsyk BG, Kityk AV, Schranz W (2005). Two-fold interferometric measurements of piezo-optic constants: Application to β-BaB2O4 crystals. Opt. Laser Technol..

[18] Landolt-Börnstein (1986). Landolt-Börnstein Tables.

[19] Maslen EN, Streltsov VA, Streltsova NR (1993). X-ray study of the electron-density in calcite, CaCO_3_. Acta Crystallogr. B.

[20] Valenzano L, Torres FJ, Klaus D, Pascale F, Zicovich-Wilson CM, Dovesi R (2006). Ab initio study of the vibrational spectrum and related properties of crystalline compounds; the case of CaCO_3_ calcite. Z. Phys. Chem..

[21] Lustemberg PG, Plessow PN, Wang Y, Yang C, Nefedov A, Studt F (2020). Vibrational frequencies of cerium-oxide-bound CO: A challenge for conventional DFT methods. Phys. Rev. Lett..

[22] Ulian G, Valdrè G (2018). Second-order elastic constants of hexagonal hydroxylapatite (P6_3_) from ab initio quantum mechanics: Comparison between DFT functionals and basis sets. Int. J. Quantum Chem..

[23] Erba A, Dovesi R (2013). Photoelasticity of crystals from theoretical simulations. Phys. Rev. B.

[24] Lide DR (2004). CRC Handbook of Chemistry and Physics.

[25] Valenzano L, Noel Y, Orlando R, Zicovich-Wilson CM, Ferrero M, Dovesi R (2007). Ab initio vibrational spectra and dielectric properties of carbonates: magnesite, calcite and dolomite. Theor. Chem. Acc..

[26] Dandekar DP (1968). Elastic constants of calcite. J. Appl. Phys..

[27] Chen CC, Lin CC, Liu LG, Sinogeikin SV, Bass JD (2001). Elasticity of single-crystal calcite and rhodochrosite by Brillouin spectroscopy. Am. Miner..

[28] Zhang GX, Reilly AM, Tkatchenko A, Scheffler M (2018). Performance of various density-functional approximations for cohesive properties of 64 bulk solids. New J. Phys..

[29] Ulian G, Tosoni S, Valdrè G (2013). Comparison between Gaussian-type orbitals and plane wave ab initio density functional theory modeling of layer silicates: Talc Mg_3_Si_4_O_10_(OH)_2_ as model system. J. Chem. Phys..

[30] Mytsyk B (2003). Methods for the studies of the piezo-optical effect in crystals and the analysis of experimental data: I. Methodology for the studies of piezo-optical effec. Ukrainian J. Phys. Opt..

[31] Tear, G. R., Chapman, D. J., Eakins, D. E., Proud, W. G. *Birefringence Measurements in Single Crystal Sapphire and Calcite Shocked Along the a Axis*. (American Institute of Physics Inc., 2017).

[32] Tear, G. R. & Proud, W. G. *Predicting the Optical Behaviour of Shock Compressed Dielectrics*. (American Institute of Physics Inc., 2017).

[33] Dovesi R, Erba A, Orlando R, Zicovich-Wilson CM, Civalleri B, Maschio L (2018). Quantum-mechanical condensed matter simulations with CRYSTAL. Wires Comput. Mol. Sci..

[34] Perdew JP, Burke K, Ernzerhof M (1996). Generalized gradient approximation made simple. Phys. Rev. Lett..

[35] Becke AD (1993). A new mixing of hartree-fock and local density-functional theories. J. Chem. Phys..

[36] Lee CT, Yang WT, Parr RG (1988). Development of the Colle-Salvetti correlation-energy formula into a functional of the electron-density. Phys. Rev. B.

[37] Grimme S (2006). Semiempirical GGA-type density functional constructed with a long-range dispersion correction. J. Comput. Chem..

[38] Civalleri B, Zicovich-Wilson CM, Valenzano L, Ugliengo P (2008). B3LYP augmented with an empirical dispersion term (B3LYP-D*) as applied to molecular crystals. CrystEngComm.

[39] Ferrero M, Rerat M, Kirtman B, Dovesi R (2008). Calculation of first and second static hyperpolarizabilities of one- to three-dimensional periodic compounds: Implementation in the CRYSTAL code. J. Chem. Phys..

[40] Ferrero M, Rerat M, Orlando R, Dovesi R (2008). The calculation of static polarizabilities of 1–3D periodic compounds: The implementation in the CRYSTAL code. J. Comput. Chem..

[41] Ferrero M, Rerat M, Orlando R, Dovesi R (2008). Coupled perturbed Hartree-Fock for periodic systems: The role of symmetry and related computational aspects. J. Chem. Phys..

[42] Kirtman B, Champagne B, Gu FL, Bishop DM (2002). Coupled-perturbed Hartree-Fock treatment of infinite periodic systems: Application to static polarizabilities and hyperpolarizabilities of polydiacetylene, polybutatriene, and interacting pairs of polyacetylene chains. Int. J. Quantum Chem..

[43] Gu FL, Otto P, Ladik J (1997). Calculation of frequency-dependent polarizabilities of quasi-one-dimensional systems. J. Mol. Model.

[44] Maschio L, Rérat M, Kirtman B, Dovesi R (2015). Calculation of the dynamic first electronic hyperpolarizability β (- ω σ; ω 1, ω 2) of periodic systems: Theory, validation, and application to multi-layer MoS2. J. Chem. Phys..

[45] Ferrero M, Rerat M, Orlando R, Dovesi R, Bush IJ (2008). Coupled perturbed Kohn-Sham calculation of static polarizabilities of periodic compounds. J. Phys. Conf. Ser..

[46] Otto P, Gu FL, Ladik J (1999). Calculation of ab initio dynamic hyperpolarizabilities of polymers. J. Chem. Phys..

[47] Perger WF, Criswell J, Civalleri B, Dovesi R (2009). Ab-initio calculation of elastic constants of crystalline systems with the CRYSTAL code. Comput. Phys. Commun..

[48] Anderson OL (1995). Equation of State of Solids for Geophysics and Ceramic Science.

[49] Erba A (2014). On combining temperature and pressure effects on structural properties of crystals with standard ab initio techniques. J. Chem. Phys..

[50] Ulian G, Valdrè G (2019). Thermomechanical, electronic and thermodynamic properties of ZnS cubic polymorphs: An ab initio investigation on the zinc-blende—rock-salt phase transition. Acta Crystallogr. B.

[51] Destefanis M, Ravoux C, Cossard A, Erba A (2019). Thermo-elasticity of materials from quasi-harmonic calculations. Minerals.

